# Supplementary material for: Do statistical heterogeneity methods impact the results of meta- analyses? A meta epidemiological study
Source: PLoS One. 2024 Mar 19;19(3):e0298526. doi: 10.1371/journal.pone.0298526 (PMC10950254; doi:10.1371/journal.pone.0298526)
Supplement: S1 Table — (DOCX) [file pone.0298526.s001.docx]

S1 Table: search strategy in PubMed

| **1** | exp orthodontic/ | 686 |
| --- | --- | --- |
| **2** | Orthodontics | 95,519 |
| **3** | Orthodontics AND braces | 535 |
| **4** | Orthodontics AND (diagnosis OR treatment) | 67,686 |
| **5** | Orthodontics AND (appliances OR techniques) | 66,183 |
| **6** | Orthodontics AND complications | 7,976 |
| **7** | ("Dentofacial Orthopedics" OR "Malocclusion") AND Orthodontics | 26,579 |
| **8** | orthodontics [MeSH Terms] | 56,058 |
| **9** | "Orthodontic Brackets"[Mesh] OR "Orthodontic Wires"[Mesh] OR "Orthodontic Appliances"[Mesh] OR "Orthodontic Space Closure"[Mesh] OR "Orthodontic Appliances, Functional"[Mesh] OR "Orthodontic Appliance Design"[Mesh] OR "Orthodontic Friction"[Mesh] OR "Orthodontic Anchorage Procedures"[Mesh] OR "Orthodontic Retainers"[Mesh] | 25,582 |
| **10** | (((((("Orthodontic Brackets"[Mesh] OR "Orthodontic Wires"[Mesh] OR "Orthodontic Appliances"[Mesh] OR "Orthodontic Space Closure"[Mesh] OR "Orthodontic Appliances, Functional"[Mesh] OR "Orthodontic Appliance Design"[Mesh] OR "Orthodontic Friction"[Mesh] OR "Orthodontic Anchorage Procedures"[Mesh] OR "Orthodontic Retainers"[Mesh]) OR (Orthodontics AND complications)) OR (Orthodontics AND (appliances OR techniques))) OR (Orthodontics AND (diagnosis OR treatment))) OR (Orthodontics AND braces)) OR (Orthodontics)) OR (exp orthodontic/) | 95,519 |
| **11** | "Malocclusion"[Mesh] OR "Malocclusion, Angle Class III"[Mesh] OR "Malocclusion, Angle Class II"[Mesh] OR "Malocclusion, Angle Class I"[Mesh] | 35,440 |
| **12** | ("Malocclusion"[Mesh] OR "Malocclusion, Angle Class III"[Mesh] OR "Malocclusion, Angle Class II"[Mesh] OR "Malocclusion, Angle Class I"[Mesh]) OR ((((((("Orthodontic Brackets"[Mesh] OR "Orthodontic Wires"[Mesh] OR "Orthodontic Appliances"[Mesh] OR "Orthodontic Space Closure"[Mesh] OR "Orthodontic Appliances, Functional"[Mesh] OR "Orthodontic Appliance Design"[Mesh] OR "Orthodontic Friction"[Mesh] OR "Orthodontic Anchorage Procedures"[Mesh] OR "Orthodontic Retainers"[Mesh]) OR (Orthodontics AND complications)) OR (Orthodontics AND (appliances OR techniques))) OR (Orthodontics AND (diagnosis OR treatment))) OR (Orthodontics AND braces)) OR (Orthodontics)) OR (exp orthodontic/)) | 108,259 |
| **13** | ("Malocclusion"[Mesh] OR "Malocclusion, Angle Class III"[Mesh] OR "Malocclusion, Angle Class II"[Mesh] OR "Malocclusion, Angle Class I"[Mesh]) OR ((((((("Orthodontic Brackets"[Mesh] OR "Orthodontic Wires"[Mesh] OR "Orthodontic Appliances"[Mesh] OR "Orthodontic Space Closure"[Mesh] OR "Orthodontic Appliances, Functional"[Mesh] OR "Orthodontic Appliance Design"[Mesh] OR "Orthodontic Friction"[Mesh] OR "Orthodontic Anchorage Procedures"[Mesh] OR "Orthodontic Retainers"[Mesh]) OR (Orthodontics AND complications)) OR (Orthodontics AND (appliances OR techniques))) OR (Orthodontics AND (diagnosis OR treatment))) OR (Orthodontics AND braces)) OR (Orthodontics)) OR (exp orthodontic/)) | 2,229 |
| **14** | ("Malocclusion"[Mesh] OR "Malocclusion, Angle Class III"[Mesh] OR "Malocclusion, Angle Class II"[Mesh] OR "Malocclusion, Angle Class I"[Mesh]) OR ((((((("Orthodontic Brackets"[Mesh] OR "Orthodontic Wires"[Mesh] OR "Orthodontic Appliances"[Mesh] OR "Orthodontic Space Closure"[Mesh] OR "Orthodontic Appliances, Functional"[Mesh] OR "Orthodontic Appliance Design"[Mesh] OR "Orthodontic Friction"[Mesh] OR "Orthodontic Anchorage Procedures"[Mesh] OR "Orthodontic Retainers"[Mesh]) OR (Orthodontics AND complications)) OR (Orthodontics AND (appliances OR techniques))) OR (Orthodontics AND (diagnosis OR treatment))) OR (Orthodontics AND braces)) OR (Orthodontics)) OR (exp orthodontic/)) AND (systematicreview[Filter]) | 2,229 |
| **15** | ("Malocclusion"[Mesh] OR "Malocclusion, Angle Class III"[Mesh] OR "Malocclusion, Angle Class II"[Mesh] OR "Malocclusion, Angle Class I"[Mesh]) OR ((((((("Orthodontic Brackets"[Mesh] OR "Orthodontic Wires"[Mesh] OR "Orthodontic Appliances"[Mesh] OR "Orthodontic Space Closure"[Mesh] OR "Orthodontic Appliances, Functional"[Mesh] OR "Orthodontic Appliance Design"[Mesh] OR "Orthodontic Friction"[Mesh] OR "Orthodontic Anchorage Procedures"[Mesh] OR "Orthodontic Retainers"[Mesh]) OR (Orthodontics AND complications)) OR (Orthodontics AND (appliances OR techniques))) OR (Orthodontics AND (diagnosis OR treatment))) OR (Orthodontics AND braces)) OR (Orthodontics)) OR (exp orthodontic/)) AND (systematicreview[Filter]) | 2,064 |
| **16** | ("Malocclusion"[Mesh] OR "Malocclusion, Angle Class III"[Mesh] OR "Malocclusion, Angle Class II"[Mesh] OR "Malocclusion, Angle Class I"[Mesh]) OR ((((((("Orthodontic Brackets"[Mesh] OR "Orthodontic Wires"[Mesh] OR "Orthodontic Appliances"[Mesh] OR "Orthodontic Space Closure"[Mesh] OR "Orthodontic Appliances, Functional"[Mesh] OR "Orthodontic Appliance Design"[Mesh] OR "Orthodontic Friction"[Mesh] OR "Orthodontic Anchorage Procedures"[Mesh] OR "Orthodontic Retainers"[Mesh]) OR (Orthodontics AND complications)) OR (Orthodontics AND (appliances OR techniques))) OR (Orthodontics AND (diagnosis OR treatment))) OR (Orthodontics AND braces)) OR (Orthodontics)) OR (exp orthodontic/)) AND (systematicreview[Filter]) | 1,322 |
| **17** | ("Malocclusion"[Mesh] OR "Malocclusion, Angle Class III"[Mesh] OR "Malocclusion, Angle Class II"[Mesh] OR "Malocclusion, Angle Class I"[Mesh]) OR ((((((("Orthodontic Brackets"[Mesh] OR "Orthodontic Wires"[Mesh] OR "Orthodontic Appliances"[Mesh] OR "Orthodontic Space Closure"[Mesh] OR "Orthodontic Appliances, Functional"[Mesh] OR "Orthodontic Appliance Design"[Mesh] OR "Orthodontic Friction"[Mesh] OR "Orthodontic Anchorage Procedures"[Mesh] OR "Orthodontic Retainers"[Mesh]) OR (Orthodontics AND complications)) OR (Orthodontics AND (appliances OR techniques))) OR (Orthodontics AND (diagnosis OR treatment))) OR (Orthodontics AND braces)) OR (Orthodontics)) OR (exp orthodontic/)) AND (systematicreview[Filter]) | 892 |
