# Supplementary material for: Do statistical heterogeneity methods impact the results of meta- analyses? A meta epidemiological study
Source: PLoS One. 2024 Mar 19;19(3):e0298526. doi: 10.1371/journal.pone.0298526 (PMC10950254; doi:10.1371/journal.pone.0298526)
Supplement: S2 Table — (DOCX) [file pone.0298526.s002.docx]

S2 Table: Excluded studies with reason.

|  | **inclusion** | **reason** | **Title** |
| --- | --- | --- | --- |
| **full** | **no** | **more than 4** | Do malocclusion and orthodontic treatment impact oral health? A systematic review and meta-analysis |
| **full** | **no** | **NA data** | Duration of tooth alignment with fixed appliances: A systematic review and meta-analysis |
| **full** | **no** | **NA data** | Complications, impacts, and success rates of different approaches to treatment of Class II malocclusion in adolescents: A systematic review and meta-analysis |
| **full** | **yes** |  | Pharyngeal airway dimensional changes after orthodontic treatment with premolar extractions: A systematic review with meta-analysis |
| **full** | **no** | **scoping review** | Dental changes in humans with untreated normal occlusion throughout lifetime: A systematic scoping review |
| **full** | **yes** |  | The influence of mobile applications and social media-based interventions in producing behavior change among orthodontic patients: A systematic review and meta-analysis |
| **full** | **yes** |  | Duration of canine retraction with fixed appliances: A systematic review and meta-analysis |
| **Abstract** | **no** | **no meta** | The effects of fixed orthodontic retainers on periodontal health: A systematic review |
| **full** | **yes** |  | Powered vs manual tooth brushing in patients with fixed orthodontic appliances: A systematic review and meta-analysis |
| **title** | **no** | **no meta** | The effectiveness of adjuncts or alternatives to the use of orthodontic retainers in preserving posttreatment outcomes: A systematic review |
| **full** | **yes** |  | Effects of bonded spurs, fixed and removable palatal crib in the early treatment of anterior open bite: A systematic review and meta-analysis |
| **full** | **yes** |  | The effect of micro-osteoperforations on the rate of orthodontic tooth movement: A systematic review and meta-analysis |
| **title** | **no** | **no meta** | Effects of lip bumper therapy on the mandibular arch dimensions of children and adolescents: A systematic review |
| **title** | **no** | **not SR** | Orthodontically induced root resorption: A critical analysis of finite element studies' input and output |
| **title** | **no** | **no meta** | Superimposition of serial 3-dimensional facial photographs to assess changes over time: A systematic review |
| **full** | **no** | **NA data** | Interradicular sites and cortical bone thickness for miniscrew insertion: A systematic review with meta-analysis |
| **full** | **yes** |  | Proximal enamel thickness of the permanent teeth: A systematic review and meta-analysis |
| **full** | **yes** |  | Analysis of the effectiveness of the fiber-reinforced composite lingual retainer: A systematic review and meta-analysis |
| **full** | **yes** |  | Effectiveness of miniscrew-supported maxillary incisor intrusion in deep-bite correction: A systematic review and meta-analysis |
| **title** | **no** |  | Lower fixed retainers: bonded on all teeth or only on canines? A systematic review |
| **full** | **yes** |  | A comparison of the effects of Forsus appliances with and without temporary anchorage devices for skeletal Class II malocclusion |
| **full** | **yes** |  | Effect of surface treatment on the mechanical stability of orthodontic miniscrews |
| **Abstract** | **no** | **no meta** | Prevalence of mandibular asymmetry in different skeletal sagittal patterns |
| **Abstract** | **no** | **no meta** | Effects of rapid maxillary expansion on temporomandibular joints |
| **full** | **no** | **no meta** | Effect of surgery-first orthognathic approach on oral health-related quality of life |
| **Abstract** | **no** | **no meta** | Effect of pharmacological agents on relapse following orthodontic tooth movement |
| **Abstract** | **no** | **no meta** | The effectiveness of locally injected platelet-rich plasma on orthodontic tooth movement acceleration |
| **full** | **yes** |  | Treatment outcome with orthodontic aligners and fixed appliances: a systematic review with meta-analyses |
| **title** | **no** | **overview** | Orthodontic treatment and root resorption: an overview of systematic reviews |
| **full** | **yes** |  | Orthodontic space closure in sliding mechanics: a systematic review and meta-analysis |
| **full** | **no** | **more than 4** | Efficacy of Miniscrew-Assisted Rapid Palatal Expansion (MARPE) in late adolescents and adults: a systematic review and meta-analysis |
| **full** | **no** | **more than 4** | Comparison of external apical root resorption with clear aligners and pre-adjusted edgewise appliances in non-extraction cases: a systematic review and meta-analysis |
| **full** | **yes** |  | Evaluation of root resorption following orthodontic intrusion: a systematic review and meta-analysis |
| **full** | **no** | **more than 4** | Mandibular propulsion appliance for adults with Class II malocclusion: a systematic review and meta-analysis |
| **full** | **no** | **no meta** | Surgically accelerated orthodontic techniques and periodontal response: a systematic review |
| **full** | **no** | **no meta** | Treatment stability with bonded versus vacuum-formed retainers: a systematic review of randomized clinical trials |
| **full** | **no** | **more than 4** | The effect of orthodontic treatment on facial attractiveness: a systematic review and meta-analysis |
| **full** | **no** | **no meta** | Social media and orthodontic treatment from the patient's perspective: a systematic review |
| **abstract** | **no** | **no meta** | Cost-effectiveness of orthodontics: a systematic review |
| **full** | **yes** |  | A non-cephalometric three-dimensional appraisal of soft tissue changes by functional appliances in orthodontics: a systematic review and meta-analysis |
| **Abstract** | **no** | **no meta** | The biological sex lens on early orthopaedic treatment duration and outcomes in Class III orthodontic patients: a systematic review |
| **full** | **yes** |  | Effectiveness of removable appliances with temperature sensors in orthodontic patients: a systematic review and meta-analysis |
| **full** | **no** | **NA data** | Impacts of using orthodontic appliances on the quality of life of children and adolescents: systematic review and meta-analysis |
| **full** | **yes** |  | Comparison between conventional and piezocision-assisted orthodontics in relieving anterior crowding: a systematic review and meta-analysis |
| **Title/Abstract** | **no** | **no meta** | Long-term dental stability after orthognathic surgery: a systematic review |
| **full** | **yes** |  | A non-cephalometric two-dimensional appraisal of soft tissue changes by functional therapy in Class II patients: a systematic review and meta-analysis |
| **full** | **yes** |  | Effectiveness of prefabricated myofunctional appliances in the treatment of Class II division 1 malocclusion: a systematic review |
| **full** | **no** | **more than 4** | Influence of clinical and demographic factors on the oral health-related quality of life of patients with cleft lip and palate undergoing orthodontic treatment: a systematic review and meta-analysis |
| **full** | **no** | **more than 5** | The relationship between malocclusion and oral health-related quality of life among adolescents: a systematic literature review and meta-analysis |
| **full** | **yes** |  | Treatment effect of bone-anchored maxillary protraction in growing patients compared to controls: a systematic review with meta-analysis |
| **full** | **yes** |  | Comparison of self-etch primers with conventional acid-etch technique for bonding brackets in orthodontics: a systematic review and meta-analysis |
| **full** | **yes** |  | Evaluation of patient-reported outcome measures (PROMs) during surgically-assisted acceleration of orthodontic treatment: a systematic review and meta-analysis |
| **full** | **yes** |  | Three-dimensional changes of the upper airway in patients with Class II malocclusion treated with functional appliances: a systematic review and meta-analysis |
| **full** | **no** | **guidelines** | Development of a clinical practice guideline for orthodontically induced external apical root resorption |
| **full** | **no** | **more than 4** | Effect of micro-osteoperforation on the rate of orthodontic tooth movement-a systematic review and a meta-analysis |
| **full** | **yes** |  | Is there variation in the depth of the curve of Spee in individuals with different dentoskeletal patterns? A systematic review with meta-analysis |
| **Title/Abstract** | **no** | **no meta** | Effects of systematic bisphosphonate use in patients under orthodontic treatment: a systematic review |
| **Title/Abstract** | **no** | **no meta** | Radiographic and histological assessment of root resorption associated with conventional and mini-screw assisted rapid palatal expansion: a systematic review |
| **Title/Abstract** | **no** | **not SR** | A tool for assessment of risk of bias in studies of adverse effects of orthodontic treatment applied in a systematic review on external root resorption |
| **full** | **yes** |  | The effect of facemask in patients with unilateral cleft lip and palate: a systematic review and meta-analysis |
| **full** | **no** | **more than 4** | Influence of low-level-laser therapy on the stability of orthodontic mini-screw implants. A systematic review and meta-analysis |
| **Title/Abstract** | **no** | **no meta** | Does medication administration affect the rate of orthodontic tooth movement and root resorption development in humans? A systematic review |
| **full** | **no** | **more than 4** | Effect of ovariectomy-induced osteoporosis on the amount of orthodontic tooth movement: a systematic review of animal studies |
| **full** | **no** | **more than 5** | Vertical stability of different orthognathic treatments for correcting skeletal anterior open bite: a systematic review and meta-analysis |
| **Title/Abstract** | **no** | **no meta** | Assessment of techniques used for superimposition of maxillary and mandibular 3D surface models to evaluate tooth movement: a systematic review |
| **full** | **yes** |  | Upper airway volumetric changes of obstructive sleep apnoea patients treated with oral appliances: a systematic review and meta-analysis |
| **full** | **no** | **more than 4** | Skeletal and dental effects of surgically assisted rapid palatal expansion: a systematic review of randomized controlled trials |
| **full** | **yes** |  | Dento-skeletal effects produced by rapid versus slow maxillary expansion using fixed jackscrew expanders: a systematic review and meta-analysis |
| **full** | **no** | **more than 4** | Assessment of the reliability of measurements taken on digital orthodontic models obtained from scans of plaster models in laboratory scanners. A systematic review and meta-analysis |
| **full** | **yes** |  | Impact of orthodontic-surgical treatment on quality of life: a meta-analysis |
| **Title/Abstract** | **no** | **no meta** | Patient-reported experiences and preferences with intraoral scanners: a systematic review |
| **Title/Abstract** | **no** | **no meta** | Reliability of cephalometric superimposition for the assessment of craniofacial changes: a systematic review |
| **full** | **no** | **prportion effect** | Comparison of long-term skeletal stability following maxillary advancement using rigid external distraction in growing and non-growing patients with cleft lip and palate: a systematic review and meta-analysis |
| **full** | **no** | **NA data** | Chronological age range estimation of cervical vertebral maturation using Baccetti method: a systematic review and meta-analysis |
| **Title/Abstract** | **no** | **no meta** | Complications reported with the use of orthodontic miniscrews: A systematic review |
| **full** | **no** | **network meta** | Clinical effectiveness of different types of bone-anchored maxillary protraction devices for skeletal Class III malocclusion: Systematic review and network meta-analysis |
| full | **yes** |  | Effect and stability of miniscrew-assisted rapid palatal expansion: A systematic review and meta-analysis |
| **full** | **no** | **more than 4** | Periodontal parameters in orthodontically tractioned teeth: A systematic review and meta-analysis |
| **full** | **no** | **no data** | Proposed parameters of optimal central incisor positioning in orthodontic treatment planning: A systematic review |
| **full** | **yes** |  | Effectiveness of miniscrew assisted rapid palatal expansion using cone beam computed tomography: A systematic review and meta-analysis |
| **full** | **no** | **no data** | Short-term treatment effects produced by rapid maxillary expansion evaluated with computed tomography: A systematic review with meta-analysis |
| **title** | **no** | **no meta** | The global distribution of permanent canine hypodontia: A systematic review |
| **Title/Abstract** | **no** | **no meta** | Effects of interproximal enamel reduction techniques used for orthodontics: A systematic review |
| **title** | **no** | **scoping review** | Artificial intelligence in orthodontics: Where are we now? A scoping review |
| **Title/Abstract** | **no** | **no meta** | Effectiveness of clear aligner therapy for orthodontic treatment: A systematic review |
| **full** | **no** | **no meta** | Interventions for impacted maxillary canines: A systematic review of the relationship between initial canine position and treatment outcome |
| **full** | **no** | **no meta** | Effect of clear aligners on oral health-related quality of life: A systematic review |
| **full** | **no** | **more than 4** | Distalization of maxillary molars using temporary skeletal anchorage devices: A systematic review and meta-analysis |
| **full** | **yes** |  | Skeletal and dental effects of Herbst appliance anchored with temporary anchorage devices: A systematic review with meta-analysis |
| **full** | **no** | **no meta** | Pulp changes from rapid maxillary expansion: A systematic review |
| **title** | **no** | **Animal** | Local hormones and growth factors to enhance orthodontic tooth movement: A systematic review of animal studies |
| **full** | **yes** |  | Alveolar bone changes in maxillary and mandibular anterior teeth during orthodontic treatment: A systematic review and meta-analysis |
| **full** | **yes** |  | Stability of single-jaw vs two-jaw surgery following the correction of skeletal class III malocclusion: A systematic review and meta-analysis |
| **full** | **yes** |  | Skeletally anchored forsus fatigue resistant device for correction of Class II malocclusions-A systematic review and meta-analysis |
| **full** | **yes** |  | Clinical effects of maxillary protraction in different stages of dentition in skeletal class III children: A systematic review and meta-analysis |
| **full** | **yes** |  | Flash-free and conventional adhesive ceramic brackets in patients undergoing orthodontic treatment: A systematic review and meta-analysis |
| **full** | **yes** |  | Incremental or maximal mandibular advancement in the treatment of class II malocclusion through functional appliances: A systematic review with meta-analysis |
| **Title/Abstract** | **no** | **no meta** | The role of muscular traction in the occurrence of skeletal relapse after advancement bilateral sagittal split osteotomy (BSSO): A systematic review |
| **Title/Abstract** | **no** | **no meta** | Influence of thyroxine supplementation on orthodontically induced tooth movement and/or inflammatory root resorption: A systematic review |
| **Title/Abstract** | **no** | **no meta** | Impact of intraoral non-pharmacological non-surgical adjunctive interventions on orthodontically induced inflammatory root resorption in humans: A systematic review |
| **full** | **no** | **network meta** | Efficacy and safety of different interventions to accelerate maxillary canine retraction following premolar extraction: A systematic review and network meta-analysis |
| **title** | **no** | **in vitro** | Comparison of shear bond strength of orthodontic brackets bonded to human teeth with and without fluorotic enamel: A systematic review and meta-analysis of experimental in vitro studies |
| **Title/Abstract** | **no** | **no meta** | The effect of ligation methods on biofilm formation in patients undergoing multi-bracketed fixed orthodontic therapy - A systematic review |
|  |  | **no access** | Is photobiomodulation effective during maxillary expansion? A systematic review and meta-analysis |
| **title** | **no** | **animal** | Could medications and biologic factors affect post-orthodontic tooth movement changes? A systematic review of animal studies |
| **full** | **yes** |  | Cortical bone thickness and bone density effects on miniscrew success rates: A systematic review and meta-analysis |
| **full** | **yes** |  | Three-dimensional analyses of short- and long-term effects of rapid maxillary expansion on nasal cavity and upper airway: A systematic review and meta-analysis |
| **Title/Abstract** | **no** | **animal** | Do analgesics used for the pain experienced after orthodontic procedures affect tooth movement rate? A systematic review based on animal studies |
| **full** | **no** | **more than 4** | Is the buccal alveolar bone less affected by mini-implant assisted rapid palatal expansion than by conventional rapid palatal expansion?-A systematic review and meta-analysis |
| **Title/Abstract** | **no** | **no meta** | Comparison of early and conventional autogenous secondary alveolar bone graft in children with cleft lip and palate: A systematic review |
| **Title/Abstract** | **no** | **no meta** | Sella turcica morphology in patients with genetic syndromes: A systematic review |
| **full** | **yes** |  | Are temporomandibular disorders associated with facial asymmetry? A systematic review and meta-analysis |
| **Title/Abstract** | **no** | **no meta** | Pain level between clear aligners and fixed appliances: a systematic review |
| **full** | **yes** |  | The effectiveness of the early orthodontic correction of functional unilateral posterior crossbite in the mixed dentition period: a systematic review and meta-analysis |
| **full** | **yes** |  | Stability of anterior open bite treatment with molar intrusion using skeletal anchorage: a systematic review and meta-analysis |
| **Title/Abstract** | **no** | **no meta** | MicroRNAs in oral fluids (saliva and gingival crevicular fluid) as biomarkers in orthodontics: systematic review and integrated bioinformatic analysis |
| **Title/Abstract** | **no** | **no meta** | Allergies/asthma and root resorption: a systematic review |
| **Title/Abstract** | **no** | **no meta** | Is there a relationship between malocclusion and bullying? A systematic review |
| **full** | **no** | **more than 4** | Duration of orthodontic treatment with fixed appliances in adolescents and adults: a systematic review with meta-analysis |
| **Title/Abstract** | **no** | **no meta** | Influence of heritability on occlusal traits: a systematic review of studies in twins |
| **full** | **no** | **more than 4** | Effects of low-level laser therapy on the orthodontic mini-implants stability: a systematic review and meta-analysis |
| **full** | **no** | **no meta** | The effect of the local administration of biological substances on the rate of orthodontic tooth movement: a systematic review of human studies |
| **full** | **no** | **more than 4** | Effects of micro-osteoperforations performed with Propel system on tooth movement, pain/quality of life, anchorage loss, and root resorption: a systematic review and meta-analysis |
| **full** | **yes** |  | Comparison of rapid versus slow maxillary expansion on patient-reported outcome measures in growing patients: a systematic review and meta-analysis |
| **title** | **no** | **methodological study** | Are orthodontic randomised controlled trials justified with a citation of an appropriate systematic review? |
